# Study protocol for a diagnostic randomized clinical trial to evaluate the effect of the use of two clinical criteria in the assessment of caries lesions around restorations in adults: the Caries Cognition and Identification in Adults (CaCIA) trial

**DOI:** 10.1186/s12903-020-01307-z

**Published:** 2020-11-10

**Authors:** Cácia Signori, Bruna Lorena Pereira Moro, Juliana Lays Stolfo Uehara, Vitor Henrique Digmayer Romero, Elenara Ferreira de Oliveira, Mariana Minatel Braga, Fausto Medeiros Mendes, Maximiliano Sérgio Cenci, CaCIA Collaborative Group, Ana Beatriz L. de Queiroz, Alessandra B. de Avila, Bruna O. Souza, Cácia Signori, Camila R. Dias, Camila T. Becker, Eduardo T. Chaves, Eugênia C. Malhão, Elenara F. de Oliveira, Juliana Lays S. Uehara, Fernanda G. da Silva, Fernanda S. da Silva, Gabriel V. Lima Kucharski, Gabriele R. dos Santos, Julia M. Torres, Karoline V. A. Pinto, Laura L. Morel, Leonardo B. Weymar, Marcelo P. Brod, Maria F. Gamborgi, Maximiliano S. Cenci

**Affiliations:** 1 Graduate Program in Dentistry, Federal University of Pelotas, Pelotas, RS Brazil; 2 Department of Pediatric Dentistry, School of Dentistry, University of São Paulo, São Paulo, Brazil

**Keywords:** Caries detection, Dental caries, Restorations, Secondary caries, Caries around restorations, Diagnosis, Visual inspection, Dental treatment, Randomized clinical trial

## Abstract

**Background:**

The assessment of restored teeth in dentistry remains a challenge, mainly related to the detection of caries around restorations. There is a diversity of clinical criteria available to assess the caries lesions, resulting in differences in the dentists’ diagnosis and treatment decisions. In addition, there is a lack of evidence regarding the best criteria to detect caries lesions around the restorations. Thus, the present protocol aims to evaluate the effect of using 2 visual criteria to assess restored teeth on the outcomes related to oral health in adults.

**Methods:**

The design protocol of the Caries Cognition and Identification in Adults trial correspond to a triple-blind randomized, controlled clinical trial with parallel-groups. Two groups will be compared: patients who will receive the diagnosis and treatment decision according to FDI (World Dental Federation) criteria—FDI group; and patients who will receive diagnosis and treatment decision according to the “Caries Associated with Restorations or Sealants” criteria defined by the International Caries Classification and Management System (ICCMS group). The participants will be followed up after 6, 12, 18, 24, and 60 months, and the restoration failure will be the primary outcome. The analysis will be conducted through Cox regression with shared frailty. The impact of oral health on quality of life and the cost-effectiveness of the methods used will be the secondary outcomes. Two-tailed analyzes will be used, considering a level of significance of 5%.

**Discussion:**

This is the first clinical trial to assess the effect of using two visual methods to detect caries lesions around restorations on the outcomes related to oral health in adults. The findings of this study will define what is the best diagnostic strategy for the assessment of caries around restorations in permanent teeth.

*Trial registration* NCT03108586 (registered 11 April 2017).

## Background

Secondary caries was recognized as one of the conditions on dentistry of the highest potential for improving future restorative treatment over the next 20 years [1]. Secondary caries is the designation given to a caries lesion adjacent to a restoration [2]. The scientific literature reports this condition as the main reason for restorations failures [2–5]. A recent review reported that the replacement of failed restorations due to secondary caries represents a high number of the restorations placed by the dentists (28.5–59% of cases). In contrast, the number of failed restorations due to secondary caries is notedly lower (2–3%) in controlled clinical trials [3, 4], which raises doubts about the real prevalence of this condition and the possibility of overtreatment. Besides, the dentists show heterogeneity in the treatment decision-making regarding secondary caries [6, 7].

The correct diagnosis of caries around the restorations is often a challenge for dentists due to aspects as the presence of gaps between the restoration and tooth surface, marginal staining, and due to the development on challenging areas of assessment, such as interproximal areas [8]. Some of these aspects can lead to an erroneous detection of caries lesion [9, 10]. Different clinical criteria have been used in the visual detection of caries around restorations [11], which may imply different interpretations about what is a secondary caries lesion. Among these criteria, two are highlighted due to the current use in research and clinic: the International Dental Federation (FDI) criteria [12] and Caries Associated with Restorations or Sealants (CARS) criteria, described in the International Caries Classification and Management System (ICCMS) [13].

Nevertheless, all studies on methods for caries detection around restorations are cross-sectional accuracy studies [11, 14]. Moreover, most studies fail to present clinical relevance and report patient-centered outcomes [11]. No randomized clinical trial has been conducted to test the best method to detect caries around restorations. Thus, we will run a randomized clinical trial to investigate the best approach to the diagnosis and decision of treatment of restorations in adults. The present protocol will aim to evaluate the effect of using 2 visual criteria, FDI and CARS criteria to assess restored teeth on the outcomes related to oral health in adults.

## Methods

### Trial design

This is a triple-blind randomized, controlled, parallel-group clinical trial. Two groups will be compared: patients who will receive the diagnosis and treatment decision according to FDI criteria [12]—FDI group; and patients who will receive diagnosis and treatment decision according to the “Caries Associated with Restorations or Sealants” (CARS) criteria from ICCMS [13]—ICCMS group. The trial—Caries Cognition and Identification in Adults (CaCIA) trial—has been registered with ClinicalTrial.gov (NCT03108586) and is currently in the active phase. The Standard Protocol Items for Clinical Trials (SPIRIT) were used to guide the present protocol as detailed in the Additional file 1: Appendix (appendix 1).

### Participants, interventions, and outcomes

#### Setting

The study will be conducted at the clinic at the School of Dentistry of the Federal University of Pelotas (UFPel). The patients (18 to 60 years old) will be randomly selected from a list of patients seeking dental treatment at the School of Dentistry.

#### Eligibility: inclusion and exclusion criteria

The inclusion criteria will consider the following:

a patients who seek dental treatment at the School of Dentistry;
b are aged 18 to 60 years;
c patients who present at least one restoration of composite resin or amalgam on a permanent posterior tooth.

The exclusion criteria will consider the following:

a patients who refuse to participate in the research;
b patients who present systemic conditions or chronic diseases that require differentiated care and follow-up. These cases will be referred to the specific services available at the School of Dentistry.
c restorations on teeth with conditions as fistula, abscess, pulp exposure, history of spontaneous dental pain, or mobility will not be included.

#### Interventions

Firstly, all patients’ dental surfaces will be examined according to the International Caries Detection and Assessment System (ICDAS) [13]. Patients meeting the inclusion criteria will be classified into subgroups. The individuals will be classified according to caries experience using the Decayed, Missing, Filled permanent Teeth (DMF-T) in 2 groups: index less or equal to 4, or index greater than 4; and also, according to the caries activity (with or without caries activity), for later block stratification.

In this first appointment, a questionnaire will be applied to assess the impact of oral health on adults’ quality of life. The instrument used will be the validated Brazilian version of the Oral Health Impact Profile-14 (OHIP-14) questionnaire [15].

**Fig. 1:**
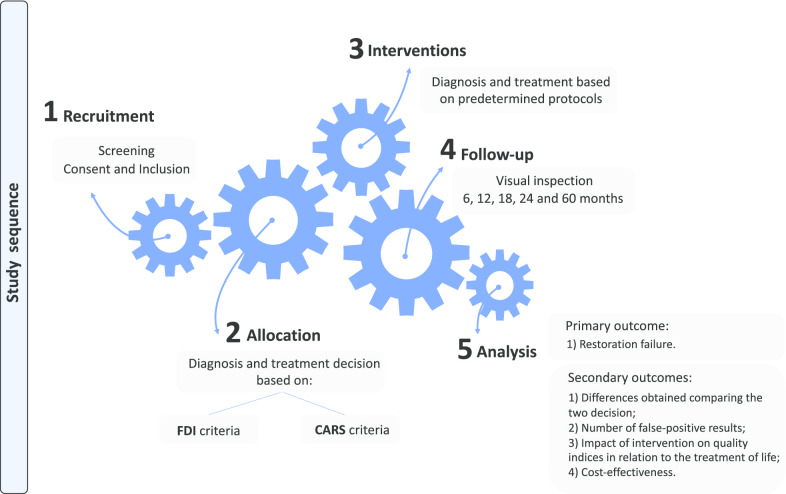
Study process

**Fig. 2:**
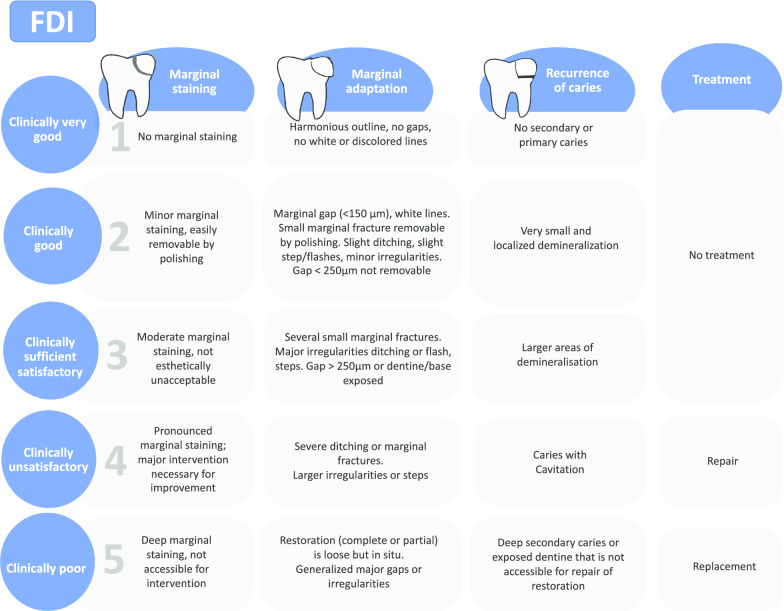
International Dental Federation (FDI) criterion linked to the treatment decision [12]

**Fig. 3:**
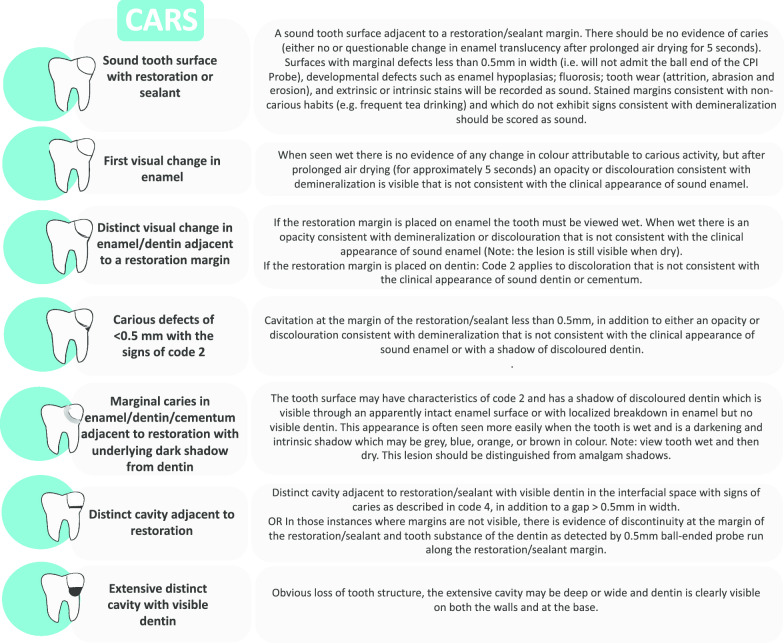
Caries-Associated with Restorations and Sealants (CARS) Detection Criteria [13]

**Fig. 4:**
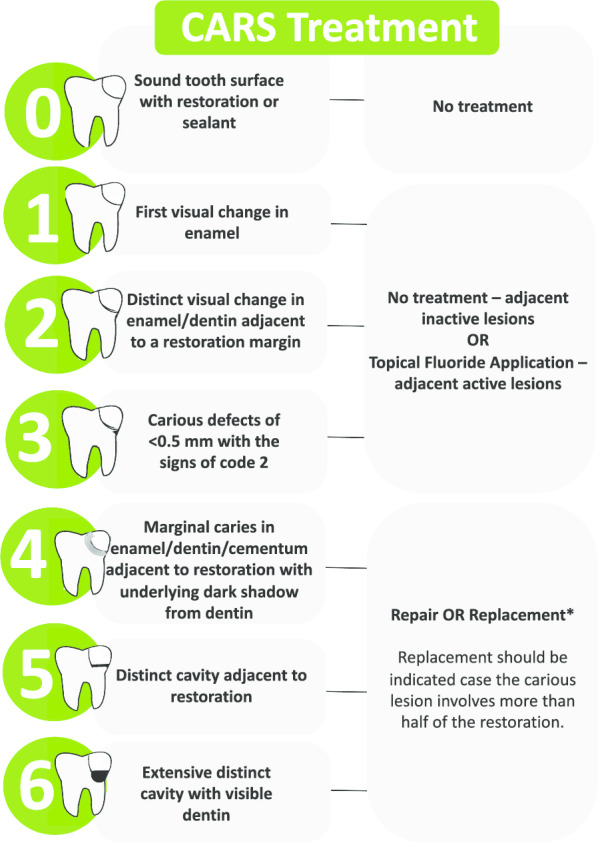
Treatment decision linked to Caries Around Restorations System—CARS adapted

The participants will be allocated into two groups (Fig. 1) according to the strategy used to diagnose and determine the treatment for caries lesions around restorations.

a *FDI group* Diagnosis and treatment decision based on the International Dental Federation (FDI) criteria (Fig. 2).
b *Experimental group* Diagnosis and treatment decision according to Caries Associated with Restorations or Sealants (CARS) detection criteria, described in the ICCMS (Figs. 3, 4).

A calibrated examiner will perform a clinical examination of the restorations. The calibration was conducted in two phases. In phase I, a series of photos on restorations with marginal defects were projected in a television in a dark room for the examiner and one expert in restorative dentistry with training and experience in the diagnosis of restorations (gold standard). The discussion of the cases was performed. Phase II was completed at the clinic; both examiner and gold standard examined a total of 20 patients, attributing the diagnosis and treatment according to FDI and CARS for each case. The answers were compared in the end, and disagreements were discussed.

In the clinical trial, after the clinical examination, the calibrated examiner will establish the treatment plan, according to the treatment indications of the criteria in which the patients were allocated. The same examiner will re-evaluate the restorations according to the other criteria. However, this procedure will only serve to future comparison among the methods. This new re-evaluation will not influence the classification and treatment proposed by the first criterion used.

The tests will be conducted in a dental chair under lighting after the teeth are cleaned with a low-rotation micromotor, rubber cup, and Robinson brush using prophylactic paste. The exams will be performed with a dental mirror and a ballpoint probe. To assess the restorations of patients allocated in the FDI group, all surfaces will be dried before evaluation [12]. For the assessment of the experimental group’s surfaces, the teeth will be evaluated wet and then dry for 5 s using the triple syringe, according to the protocol established by the ICCMS [13].

#### Dental treatment protocols

The restorations, therefore, will be submitted to the proposed treatment according to the first evaluation performed. These treatments will be performed according to predefined protocols by operators blinded to the criterion used to reach the treatment decision.

In all situations, the carious tissue, if present, will be removed, as well as the dental restorations, when indicated.

Both repair and replacement of restorations will be performed following the adhesive protocol described by the manufacturer (Adper Scotchbond Multi-Purpose, 3M ESPE, USA) to the use of resin restorations. The conventional composite resin (Filtek Z350 XT, 3M ESPE, USA) will be inserted on the cavity using increments. Besides the proposed treatment for restorations, other necessary treatments for the patient will also be performed. Additional treatments (not involving repairs/replacements) will be planned/defined by the operator responsible for the patient's initial clinical examination.

#### Follow-up visits

After completing the treatment performed at the last restoration of each participant, they will return to evaluate the outcomes after 6, 12, 18, 24, and 60 months.

The restorations will be evaluated through clinical inspection (mirror and ballpoint probe) by a previously calibrated examiner. The treatment needs will be established according to the demands of the patients. The examiner will be blind concerning previous allocation groups and previously performed treatments. If the patient needs further treatment-related or not to restorations, it will be performed.

The instrument OHIP-14 will be reapplied 1 week after the patients receive all the interventions needed, at 24 months and 60 months, to assess the impact on the long-term quality of life.

#### Outcomes

The primary outcome will be restoration failure. The secondary outcomes will be the differences obtained comparing the two indices in relation to the treatment decision, the number of false-positive results (cases initially indicated to repair or replacement, in which during the intervention no decayed tissue was found), the impact of the intervention on quality of life and cost-effectiveness.

### Participant timeline

**Fig. 5:**
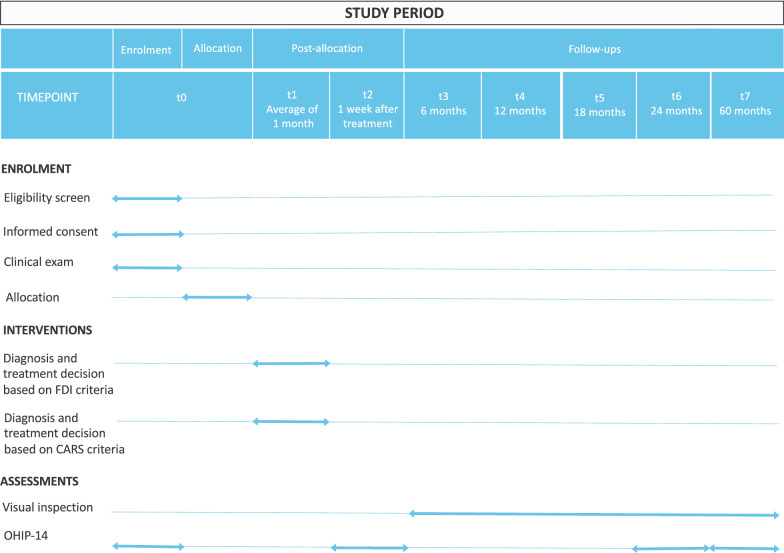
Standard protocol items: enrolment, interventions, and assessments

The study will be recruiting patients from October 2016 to February 2020. The study’s enrollment for each participant will lead approximately 61 months, estimating 1 month of treatment and 60 months of follow-up. The study phases are presented in Fig. 5.

### Sample size

The sample calculation was performed based on the primary outcome of the randomized clinical trial (percentage of restorations requiring reintervention). The calculation considered a 2-year failure rate of approximately 10% for occlusal restorations [16] and 30% for occlusal-proximal restorations [17]. It was also assumed that about 10% of the replaced restorations and 14% of the restorations undergoing repair fail again [18]. Thus, estimating that half of the sample is from occlusal restorations, an operative reintervention requirement rate of 24% was estimated in 2 years. The number of 522 restorations was reached, based on an absolute difference of 10% between the groups, using a two-tailed test. As a participant can contribute with more than one restoration, 20% was added to this value (n = 626). Thus, considering a predetermined average of inclusion of 5 teeth per patient, and adding 20% to possible sample losses, a minimum number of 152 patients was reached to be included in the trial.

### Recruitment

The recruitment will occur in the School of Dentistry, as it receives a considerable number of patients looking for dental treatment. An average of 200 patients seeks dental treatment attendance per month, totaling approximately 2400 patients per year. The patients will be aleatorily selected from this broader sample.

### Assignment of interventions

#### Allocation: sequence generation and concealment mechanism

The random list will be generated via the website (www.sealedenvelope.com). The study participants will be examined, classified according to predetermined criteria determined by the randomization stratified by blocks, and then referred to the examiner to evaluate the restorations. The strata will be: (1) DMF-T index less or equal to 4 without caries activity; (2) DMF-T index less or equal to 4 with caries activity; (3) DMF-T greater than 4 without caries activity; and (4) DMF-T greater than 4 with caries activity.

To ensure allocation confidentiality, we will use opaque, sealed, and consecutively numbered envelopes. The allocated group will be revealed to the examiner before the start of the examination.

### Implementation

A clinical operator not involved with the study design or evaluation will carry out the patient's initial exam. A pre-calibrated examiner will then examine the restorations and indicate the treatments based on the criteria defined by the randomization. The responsible for the dental treatment will perform the treatments based on the patient’s treatment plan provided for them, without any access to the allocation group of the patient.

### Blinding

The patients, care providers responsible for the dental treatment (undergraduate students and graduate students), and the assessor who will evaluate the outcomes will be blind to the participants’ allocation group.

### Data collection, management, and analysis

The follow-up assessments will be performed by a pre-calibrated examiner, who does not have previous contact with the patient and with last information about the allocation groups and treatments performed. The treatment needs will be established according to the demands of the patients.

The clinical data will be registered on sheets previously organized on Microsoft Excel Software. All data, except those that might reveal the participants’ identities, will be shared in a public repository after accepting all manuscripts related to these studies.

The survival analysis will be used to analyze the primary outcome. Kaplan–Meyer graphs will be constructed, and the methods will be compared to each other with Cox regression with shared frailty. The calculation of sensitivity, specificity, and accuracy will consider the results obtained with the indices and the classification of the presence or not of caries lesion by the proposed reference standard. 95% CI values will be calculated with adjustments as one individual may have more than one restoration included, using a suggestion previously published [19]. The sensitivity, specificity, and accuracy between the methods will be compared using multilevel analysis (3 levels: assessment, tooth, and child/adults). As also, for the comparisons between the treatment decisions obtained with the different criteria. The cost-effectiveness ratio will also be verified, considering as effect the prevention of the primary outcome, as well as other secondary endpoints of interest, and the cost spent to reach such a condition with each of the indices. For all tests, two-tailed analyzes will be used, considering a level of significance of 5%. Analyzes will be performed using the statistical package Stata 15.0 (Stata Corp, College Station, USA).

### Monitoring

#### Data monitoring

Independent regulation of data collection, management, and analysis will be assumed independently by MSC.

#### Harms

The procedures performed offer minimal risk to the oral health of patients. The adverse effects are represented by the teeth with pain episodes, postoperative sensitivity, tooth fracture during the restorative procedure, teeth requiring endodontic treatment, and exodontia. In dental treatment, the possibility of occurrence of these effects is usually present.

#### Auditing

The data entered will be conducted by one of the authors of the study. The data will be weekly inspected. The inconsistencies will be verified, corrected, and registered.

### Ethics and dissemination

#### 2.11.1 Research ethics approval

This study was submitted and approved by the Ethical Committee from the School of Dentistry, Federal University of Pelotas (No. CAAE: 53463316.1.0000.5318).

#### Consent and assent

Informed consent will be provided and assigned by the participants.

#### Confidentiality

Identification numbers will be used to assure participant confidentiality during data analysis. Participants’ files will be stored in a secure room.

#### Availability of data

The datasets used and/or analysed during the current study will be available from the corresponding author (MSC) on reasonable request.

#### Ancillary and post-trial care

The participants will receive dental treatment during and after the end of the study.

#### Dissemination policy

The findings will be reported in full through national and international journals, patient newsletters, and websites.

## Discussion

The assessment of restorations in dentistry remains a challenge, even after many years of research and discussion [5, 11, 20]. The main point of debate is the detection of caries around restorations. The dentists do not show the same line of thinking about what is and what is not a caries lesion adjacent to the restoration. Also, there is a diversity of clinical criteria available to assess the caries lesions, which may influence the dentists’ different opinions and on the treatment decisions taken [6, 7, 11]. In addition, there is a lack of evidence regarding the best criterion to detect secondary caries lesions.

The studies available about caries detection methods around restorations are in general studies of accuracy with cross-sectional experimental design [11, 14]. Still, there is a limited number of studies, with most of the studies being performed in vitro, showing a high risk of bias [11]. The accuracy studies are important to investigate the validity of the diagnostic method. Still, the best methods to be used in clinical practice should not be made based solely on these studies [11, 21, 22]. Besides, most studies fail to present clinical relevance and do not investigate patient-centered outcomes [11]. It is essential to explore which methods would assure more benefits to the patient’s health [23]. And this is only possible through randomized clinical trials with proper follow-up periods.

Randomized clinical trials aiming to evaluate diagnostic tools are usual in the medical field. However, the same is not applied to dentistry, which shows a limited number of studies with this experimental design [24]. No previous study compared the accuracy of FDI and CARS criteria clinically to detect secondary caries on permanent teeth, and the impact of using the criteria on the restorative treatment decisions. It is also important to observe that in our study, the group based on the International Dental Federation (FDI) criteria included the recurrent caries criteria described by the FDI and the marginal staining and marginal adaptation criteria to complement the assessment of the restorations. This decision was based on the fact that many dentists and studies associate these two defects (marginal staining and marginal adaptation) with detecting caries lesions around the restorations [11].

The detection criteria are proposed and used to assess a particular condition and to aid in the selection of the most suitable treatment. Considering the restorative treatment, the proper treatment may range since the monitoring, repair, or replacement of the restoration [25]. Still, the correct diagnosis of caries around the restorations can lead to greater longevity of the restorative treatment, improving the patients’ oral health, and reducing treatment costs [26]. A considerable burden on health care expenditure is attributed to the operative management of restorations due to the detection of secondary caries [5]. Also, the clinical criteria used to caries detection should be in line with the current philosophy of minimally invasive dentistry [27]. Using a method that tends to overtreatment, accelerating the repetitive restorative cycle is not desirable [28].

To the best of our knowledge, this will be the first study to assess the effect of two visual methods for evaluating caries lesions around restorations on the outcomes related to oral health in adults. The hypothesis under evaluation is that there will be a difference between the interventions established considering the outcomes centered on the restoration, tooth, or the patient.

### Trial status

**Fig. 6:**
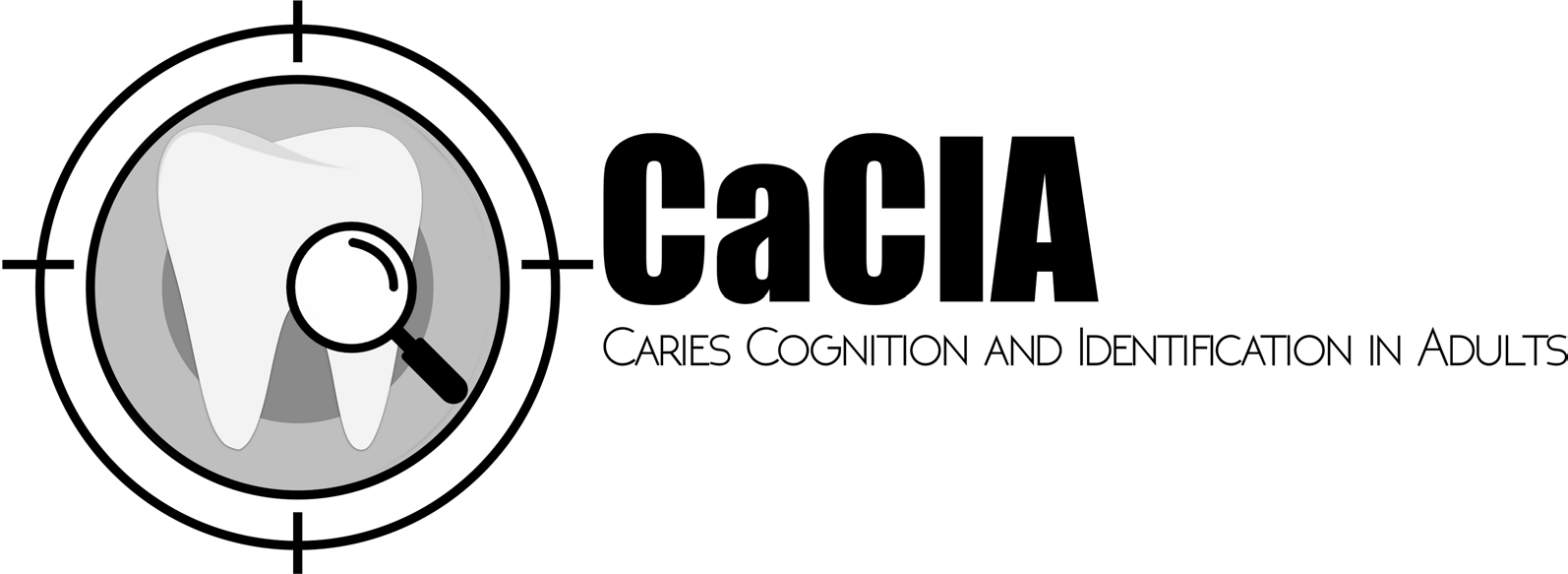
CaCIA trial logotype

The trial is recruiting participants. The recruitment has been in progress from October 2016 until now. The end of the recruitment is planned for February 2020. Figure 6 presents the CaCIA trial logotype.

## Supplementary information

**Additional file 1.** SPIRIT checklist.

## Data Availability

The dataset will be available in a public repository after the acceptance of the manuscripts.

## Abbreviations

CaCIA: Caries cognition and identification in adults
CARDEC: Caries deection in children
CARS: Caries associated with restorations or sealants
CI: Confidence interval
DMF-T: Decayed, missing, filled permanent teeth
FDI: World Dental federation
ICCMS: International Caries Classification and Management System
OHIP-14: Oral Health Impact Profile-14
SPIRIT: Standard Protocol Items for Clinical Trials
UFPEL: Federal University of Pelotas
